# Effectiveness of Web-based Interventions on Patient Empowerment: A Systematic Review and Meta-analysis

**DOI:** 10.2196/jmir.1286

**Published:** 2010-06-24

**Authors:** David Samoocha, David J Bruinvels, Nieke A Elbers, Johannes R Anema, Allard J van der Beek

**Affiliations:** ^1^Department of Public and Occupational HealthThe EMGO Institute for Health and Care ResearchVU University Medical CenterAmsterdamNetherlands; ^2^Research Center for Insurance Medicine AMC-UWV-VUmcAmsterdamNetherlands; ^3^IGER, the Amsterdam Interdisciplinary Center of Law and HealthAmsterdamNetherlands

**Keywords:** Patient empowerment, Internet, eHealth

## Abstract

**Background:**

Patient empowerment is growing in popularity and application. Due to the increasing possibilities of the Internet and eHealth, many initiatives that are aimed at empowering patients are delivered online.

**Objective:**

Our objective was to evaluate whether Web-based interventions are effective in increasing patient empowerment compared with usual care or face-to-face interventions.

**Methods:**

We performed a systematic review by searching the MEDLINE, EMBASE, and PsycINFO databases from January 1985 to January 2009 for relevant citations. From the 7096 unique citations retrieved from the search strategy, we included 14 randomized controlled trials (RCTs) that met all inclusion criteria. Pairs of review authors assessed the methodological quality of the obtained studies using the Downs and Black checklist. A meta-analysis was performed on studies that measured comparable outcomes. The GRADE approach was used to determine the level of evidence for each outcome.

**Results:**

In comparison with usual care or no care, Web-based interventions had a significant positive effect on empowerment measured with the Diabetes Empowerment Scale (2 studies, standardized mean difference [SMD] = 0.61, 95% confidence interval [CI] 0.29 - 0.94]), on self-efficacy measured with disease-specific self-efficacy scales (9 studies, SMD = 0.23, 95% CI 0.12 - 0.33), and on mastery measured with the Pearlin Mastery Scale (1 study, mean difference [MD] = 2.95, 95% CI 1.66 - 4.24). No effects were found for self-efficacy measured with general self-efficacy scales (3 studies, SMD = 0.05, 95% CI -0.25 to 0.35) or for self-esteem measured with the Rosenberg Self-Esteem Scale (1 study, MD = -0.38, 95% CI -2.45 to 1.69). Furthermore, when comparing Web-based interventions with face-to-face deliveries of the same interventions, no significant (beneficial or harmful) effects were found for mastery (1 study, MD = 1.20, 95% CI -1.73 to 4.13) and self-esteem (1 study, MD = -0.10, 95% CI -0.45 to 0.25).

**Conclusions:**

Web-based interventions showed positive effects on empowerment measured with the Diabetes Empowerment Scale, disease-specific self-efficacy scales and the Pearlin Mastery Scale. Because of the low quality of evidence we found, the results should be interpreted with caution. The clinical relevance of the findings can be questioned because the significant effects we found were, in general, small.

## Introduction

Patient empowerment refers to the enhanced ability of patients to actively understand and influence their own health status [1]. Patient empowerment focuses on control in individuals’ experience of health, disease, and illness, as well as the roles of health care organizations, communities, and the broader health care system [2].

Since its introduction to health care in the 1970s [3], the popularity of the idea of patient empowerment has emerged in the context of several significant societal trends, such as a growth in health care consumerism and, as a result, the need for governments to reduce health care costs [4]. In this case, patient empowerment potentially could be used to justify cost-cutting in which part of the responsibility for care is transferred to individual citizens [5]. Furthermore, increased patient activism and organization has led to more focus on patient empowerment initiatives [6]. Revealingly, and in line with these tendencies, the World Health Organization (WHO) has described patient empowerment as a “prerequisite for health” and “a proactive partnership and patient self-care strategy to improve health outcomes and quality of life among the chronically ill [7].”

During the last decades, the focus on empowerment resulted in many initiatives to increase patient empowerment. In general, strategies to increase patient empowerment have tended to address two aspects of patients’ experience of illness: (1) disease management and (2) relationships with health care providers [8]. An increasing number of studies have been conducted in which approaches to increasing patient empowerment have been evaluated. These approaches have varied from patient self-management programs [9-16], to promoting patient involvement in treatment decision-making [17,18], to facilitating the physician-patient interaction [19,20]. Most of these interventions have taken place face-to-face or in facilitator-led group sessions.

Although some face-to-face or group session interventions to increase patient empowerment have been found to be effective, for example, in decreasing depression [21] or in job retention among the chronically ill [22], it is believed that the real opportunities for patient empowerment started with the rise of the Internet and eHealth [23]. In recent years, the number of Internet users has increased considerably, and the Internet is being employed more frequently to locate information on health and health care delivery [24]. The latest studies have shown that, among all Internet users, an estimated 58% consult the Web for health purposes [25]. Because of this increased use of the Internet and its huge potential for delivering patient education and management programs, the Internet may have a revolutionary role in retooling the health care industry [23]. Some scientific evidence already exists on the effectiveness of the Internet to improve health outcomes [26,27], increase specified knowledge [28,29], achieve behavior change [30,31], and increase participation in health care [32]. Therefore, in recent years, a growing number of interventions aimed at patient empowerment are, not surprisingly, delivered online.

In this systematic review, we investigated whether these Web-based interventions were effective in increasing patient empowerment compared with usual care or face-to-face interventions.

## Methods

### Inclusion Criteria

#### Types of Studies

Only randomized controlled trials (RCT), quasi-randomized controlled trials, before-and-after studies, and interrupted time series analyses were included.

#### Types of Participants

Studies in which the intervention was aimed at patients or clients with a medical problem were included. Studies that included children and adolescents less than 18 years of age were excluded to create more homogeneity in the study population.

#### Types of Intervention

Studies in which the treatment consisted of a Web-based intervention were included. Web-based interventions were defined as all interactive Web applications accessed via the Internet or an intranet. Furthermore, we excluded studies if the intervention did not contain any aspects of health education or intention to change health-related behavior.

#### Types of Control Intervention

Studies were included only if the control intervention consisted of either usual care or a face-to-face intervention.

#### Types of Outcome Measures

Studies that measured empowerment or an empowerment-related component were included. Given the absence of a generally accepted definition of empowerment and conflicting views on how to measure empowerment, we decided to initially include concepts that are often linked to empowerment. Examples of these are self-efficacy, mastery, self-control, self-esteem, perceived control, perceived competence, or involvement in the decision-making process [33].

No language restriction was applied.

### Search Strategy

Publications were retrieved by a search of the following electronic databases:

- MEDLINE (l985 to January 2009)
- EMBASE (1985 to January 2009)
- PsycINFO (1985 to January 2009)

Detailed search strategies are presented in the Multimedia Appendix. Briefly, we combined two search concepts, the first consisting of the outcome measure (eg, “empowerment” or “self-efficacy”) and the second of the intervention (eg, “Internet” or “website”). Various synonyms were used for each concept. We chose a sensitive search strategy so that we would not miss any potentially relevant publications.

### Study Identification and Data Abstraction

Citations and brief records identified by the search strategy were downloaded electronically to the bibliographic management package Reference Manager 11 (Thomson Reuters, Carlsbad, CA, USA). The study selection was completed in three steps. In step 1, two reviewers (authors DS and DB) independently screened the titles, keywords, and abstracts of the studies obtained by the search strategy to determine if they met the inclusion criteria. When inclusion or exclusion of a study could not be based on the screening of the title, keywords, and abstract, in step 2, the full article was retrieved and checked for inclusion. This was again, done by two reviewers (authors DS and NE). A consensus meeting with a third reviewer (DB) was arranged to sort out disagreements between reviewers. In step 3, we searched the reference lists of the included studies to find additional publications. Additionally, from all citations that were initially identified by the search strategy, we checked all systematic reviews concerning Web-based interventions and searched in the reference lists of these reviews to find additional publications that met our inclusion criteria.

Two reviewers (DS and NE) extracted the data using a data extraction form that included information on study design, randomization level, population, follow-up period, description of the intervention and control group treatments, and data on relevant outcomes. If certain studies did not report sufficient information on the outcomes, missing data (for example, standard deviations) were calculated according to guidelines in the *Cochrane Reviewers’ Handbook* [34]. If it was not possible to calculate missing data, authors of the studies were contacted and additional information on the missing data was requested.

### Quality Assessment

We evaluated the quality of individual studies using the Downs and Black quality assessment method, which is a list of 27 criteria to evaluate both randomized and nonrandomized trials [35]. This quality assessment scale (QAS) assesses study reporting, external validity, and internal validity (ie, bias and confounding), and has been ranked in the top six quality assessment scales suitable for use in systematic reviews [36,37]. As has been done in other reviews using the Downs and Black scale [38,39], the tool was modified slightly for use in this review. Specifically, the scoring for question 27 dealing with statistical power was simplified to a choice of awarding either 1 point or 0 points depending on whether there was sufficient power to detect a clinically important effect. The criterion was that to detect a 10% difference, at least 87 subjects had completed follow-up in both the intervention and control groups of the study, assuming power of .90 and alpha of .05. The maximum score for item 5, reporting of principal confounders was 2. Downs and Black score ranges were grouped into the following 4 quality levels: excellent (26 to 28), good (20 to 25), fair (15 to 19), and poor (less than 14).

Two reviewers (DS and DB) independently assessed the quality of the included studies. A consensus method was used to resolve disagreement.

### Data Analysis and the GRADE Approach

Analyses of this review were based on the outcome measure, that is, empowerment or an empowerment-related outcome. For studies that were comparable with respect to the control intervention and the outcome, results were pooled using meta-analyses. In these analyses, we included final measurements of continuous data. We were able to do so, because all included studies in this review were RCTs or quasi-RCTs, and no study reported significant baseline differences between the intervention and the control group. Since there were many different scales or instruments used to measure the same outcome, we could not use weighted mean differences and therefore calculated standardized mean differences (SMD). SMDs obtained from the meta-analyses were then reexpressed to a familiar instrument, using a so-called back-translation technique. In this technique, an instrument is selected from a study included in the meta-analysis that is representative of the population and at a low risk of bias, and then the standard deviation of the outcome measure of the control group of this study (the end of study mean) is multiplied by the pooled SMD.

We chose a random-effects meta-analysis because it was considered a more appropriate model to combine the results of the included studies, which were clinically and methodologically diverse [40]. Additionally, publication bias was tested by inspecting the funnel plot on outcomes that were measured in more than 8 studies. A funnel plot is a scatterplot of treatment effect against a measure of study size. An asymmetric funnel indicates a relationship between treatment effect and study size, suggesting a possibility of publication bias. For all analyses Review Manager software (version 5.0) was used [41].

We present the overall quality of the evidence using the GRADE approach as recommended by the *Cochrane Handbook for Systematic Reviews of Interventions* [34] because of its many advantages [42]. That is, for each specific outcome, the quality of the evidence was based on five factors: (1) limitations of the study design or the potential for bias across all studies that measure that particular outcome, (2) consistency of results, (3) directness (generalizability), (4) precision (sufficient data), and (5) the potential for publication bias. The overall quality was considered to be high if multiple RCTs with a low risk of bias provided consistent, generalizable results for the outcome. The quality of evidence was downgraded by one level if one of the factors described above was not met. Likewise, if two or three factors were not met, we downgraded the level of evidence by two or three levels, respectively. Thus, the GRADE approach resulted in four levels of quality of evidence: high, moderate, low, and very low. In the case of only one study measuring an outcome, the data were considered to be “sparse,” and subsequently the evidence was labeled as “low quality evidence.” If only one study was present for a given comparison, the results are described in the text. GRADEprofiler software (version 3.2) was used [43].

## Results

### Study Selection

**Figure 1:**
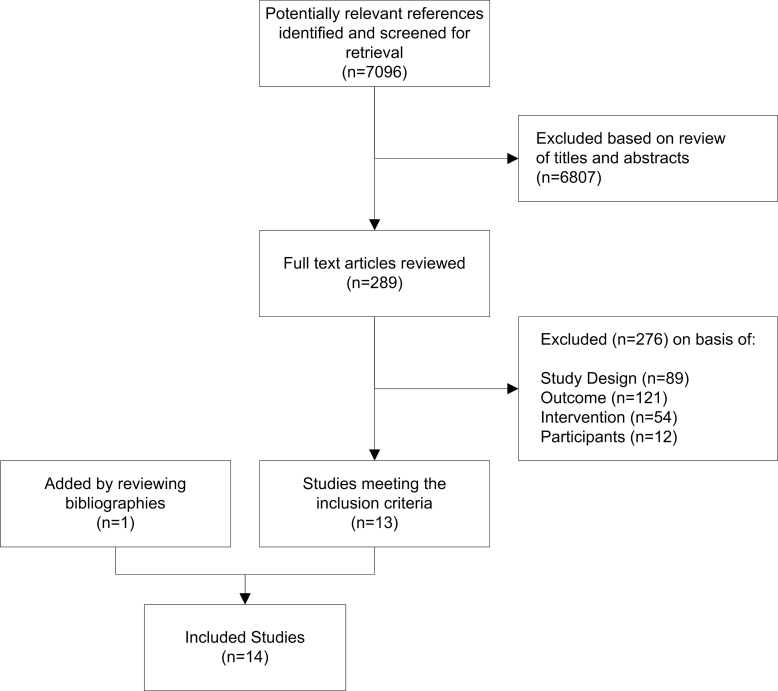
Study selection

From all databases combined, we identified a total of 7676 titles: 1823 in MEDLINE, 3540 in EMBASE and 2313 in PsychINFO. After exclusion of duplicates, DS and DB reviewed the 7096 titles and abstracts. Figure 1 shows a flow diagram of the reviewed studies.

We selected 289 publications for retrieval of full text versions. From these 289 studies, initially 18 publications met our inclusion criteria: these were publications reporting results of 16 RCTs and 2 quasi-RCTs. The main reason for excluding studies was based on the outcome criteria: the majority of the studies did not measure empowerment or an empowerment-related outcome. From the 18 studies that initially met our inclusion criteria, 2 publications of Man et al [44,45] reported on the same study and were handled as one RCT. The publication of Kukafka et al [46] was excluded because standard deviations of the outcome (self-efficacy) were not reported and were not retrieved after contacting the author. The study by Wangberg [47] did not have an appropriate contrast for the website intervention. Instead, this study compared two study groups that both used tailored Internet interventions. After closer examination, the study by Robinson [48] was excluded because it did not meet the patient criteria, and the study by Williams et al [49] was excluded because the intervention in this study was not solely Web-based. The additional reference search resulted in one extra publication [50] that met our inclusion criteria. Because this publication by Warmerdam et al contained a description of a study protocol, we contacted the authors and received relevant unpublished data. With this study included, the total number of studies included in this review was 14 [45,50-62].

### Study Characteristics

Table 1 shows the characteristics of the included studies.

**Table 1:** Characteristics of the included studies

Citation	Population/ Setting (n, % Female)	Duration of Follow-up (% dropout)	Intervention	Comparison Treatment	Outcome Measure(s) (Instrument)	Quality Assessment Score

Cousineau et al 2008 [51]	Online psycho-educational support for infertile women (190, 100%)	4 weeks (3%)	Tailored website containing in vitro fertilization (IVF)-specific cognitive behavioural skill training (CBT) and stress management. Total content: 90 minutes, available over a 4-week period^b,c,e^	Waiting list	Self-efficacy, infertility specific (Infertility Self-Efficacy Scale [51]).	26, Excellent
Gollings et al 2005 [52]	Treatment of body dissatisfaction among women 18 to 30 years old (39, 100%)	8 weeks (18%)	The Set Your Body Free Group Body Image Program: eight therapist-led, online chat sessions (weekly, session duration: 90 minutes), 24/7 discussion board^a^	Face-to-face delivery of the same intervention	Self-esteem (Rosenberg Self-esteem Scale [RSE] [67])	16, Fair
Hill et al 2006 [53]	Increasing psychological health among chronically ill, rural women(12, 100%)	12 weeks (17%)	Women-to-Women (WTW) project: an online web-based educational tool aimed at increasing Web skills, coping with chronic illness, handling family finances, etc^a,b^	No care	(1) Empowerment (adapted Diabetes Empowerment Scale [DES] [63]) (2) General self-efficacy (General Self-Efficacy [GSE] [65]) (3) Self-esteem (RSE [67])	17, Fair
Hirai and Clum 2005 [54]	Internet help for patients with posttraumatic stress disorder (36, 78%)	8 weeks (25%)	Internet-based, interactive cognitive behaviour program (8 weeks) consisting of relaxation training, mastery tests, cognitive restructuring and exposure modules^c^	Waiting list	Self-efficacy, post-traumatic syndrome specific (adapted scale)	15, Fair
Homko et al 2007 [55]	Managing underserved women with gestational diabetes mellitus in a prenatal clinic (63, 100%)	From 4 to 37 weeks, depending on gestation at inclusion (10%)	Web-based disease management interactive telemedicine system. Components: health information and education, patient electronic medical record, and communication with health team^a,b,d^	Paper logbook	Empowerment (DES [63])	18, Fair
Lorig et al 2002 [56]	Internet help for back pain patients (580, 39%)	1 year (27%)	Closed and moderated email discussion group, copy of the Back Pain Helpbook, and a videotape on how to continue active life with back pain^a^	Usual care	Self-efficacy, specific to management of back pain (adapted scale)	20, Good
Lorig et al 2006 [57]	Internet help for patients with chronic diseases (heart or lung disease and type 2 diabetes) (958, 71%)	1 year (19%)	Interactive website (6-week program) based on the book *Living a Healthy Life with Chronic Conditions.* Online workshops with Web moderator, individual exercise programs, cognitive symptom management etc^a,b,c^	Usual care	Self-efficacy, disease management specific (adapted scale)	20, Good
Lorig et al 2008 [58]	Internet-based arthritis self-management program for patients with fibromyalgia (855, 90%)	6 months (25%)	Arthritis Self-Management (6-week) Program: health education, bulletin board discussion, individual tools such as exercise logs, medication diaries, and a tailored exercise program ^a,b,c,d,e^	Usual care	Self-efficacy, arthritis specific (adapted scale)	22, Good
Man et al 2006 [44]	Problem-solving skill training for people with acquired brain injury (ABI) (59, 43%)	8 weeks (24%)	Online interactive multimedia presentations on knowledge and concepts required for persons with ABI to function independently ^c^	Waiting list	Self-efficacy, ABI specific (adapted scale)	21, Good
Nguyen et al 2008 [59]	Internet-based dyspnea self-management program for patients with chronic obstructive pulmonary disease (50, 44%)	6 months (22%)	Website containing structured education for dyspnea management strategies, skills training, peer interaction, symptom and exercise monitoring, and exercise consultations (6- month program)^a,b,c,d^	Face-to-face delivery of the same intervention	Mastery, subscale of the Chronic Respiratory Questionnaire (CRQ [59])	20, Good
Ross et al 2004 [60]	Web-based online medical record for patients with congestive heart failure in a speciality clinic (107, 23%)	6 months (22%)	SPPARO web interface, providing patients with a medical record, educational guide and a messaging system^a,b,d^	Usual care	Self-efficacy, patient specific (adapted scale)	23, Good
Tuil et al 2007 [61]	Internet-based personal health record for patients undergoing IVF and intracytoplasmic sperm injection (ICSI) treatment in an academic research environment (244, 50%)	16 weeks (26%)	IVF educational interactive website consisting of general information, a personal medical record with tailored clarifications, and communication (forum, email, chat)^a,b,d,e^	Usual care	(1) General self-efficacy (GES [64]) (2) Self-efficacy, IVF specific (adapted scale)	18, Fair
Warmerdam et al 2009 [50]	Internet treatment for adults with depressive symptoms (263, 71%)	Intervention 1:12 weeks (46%) Intervention 2: 5 weeks (42%)	Intervention 1: Cognitive Behavioral Therapy (12 weeks) based on changing patients’ cognitive patterns^c^ Intervention 2: Problem-Solving Treatment (5 weeks) aiming at controlling practical problems patients face^c^	Waiting list	Mastery (Pearlin Mastery Scale [66])	23, Good
Zutz et al 2007 [62]	Website for potential participants for hospital-based cardiac rehabilitation programs (15, 20%)	12 weeks (13%)	Website with the ability to interactively monitor heart rate and blood pressure. Plus scheduled one-on-one chat sessions with program nurse case manager, weekly education sessions and monthly ask-an-expert group chat^a,b,d^	Usual care	(1) General Self-efficacy (GSE [65]) (2) Self-efficacy; exercise specific (adapted scale)	15, Fair

Web-based intervention contains: ^a^ communication options, such as a forum, chat or (moderated) discussion board

^b^ health information to increase knowledge

^c^ disease specific self-management modules

^d^ e-monitoring, such as a patient medical records or symptom diaries

^e^ tailored messages or information

#### Design of the Included Studies

Of the 14 included studies, 13 were RCTs, while the study that was reported in two publications [44,45] was a quasi-RCT. Most studies compared two study groups: an intervention group in which the treatment was a Web-based intervention and a control group receiving usual care or no care. Exceptions were the studies of Nguyen et al [59] and Gollings et al [52] in which comparisons were made between a Web-based intervention and a face-to-face intervention. Furthermore, the study by Man et al [45] consisted of four intervention arms: a control group, a computer-assisted training program, an online training program, and a therapist-administered training program. For this review, we compared the online training program with the control group. Also, the study by Warmerdam et al [50] included three interventions arms: a Web-based cognitive behavior program, a web-based problem-solving program, and a control group.

The duration of the follow-up measurements varied from 8 weeks [54] up to one year [56,57]. Also, many differences were found in the exact content of the Web-based interventions.

#### Participants

The number of participants varied from 15 [62] to 958 [57]. Studies differed regarding patient groups. For example, various studies included infertility patients, patients with post-traumatic stress disorder, patients with diabetes, or back pain patients. The mean dropout rate was 23% (SD 11%) after an average follow-up duration of 19 weeks (SD 15 weeks). Participants’ compliance with the intervention was not clearly described in many of the included studies.

#### Outcomes

Empowerment was explicitly measured in only two studies. Both of these used the Diabetes Empowerment Scale (DES) [63]. Although the DES is meant to measure diabetes-related empowerment, the study of Hill et al [53] showed that the DES can be adapted to other diseases. All other included studies (one or more) measured empowerment-related outcomes: 9 studies measured disease-specific self-efficacy, 3 measured general self-efficacy, 2 measured mastery, and 2 measured self-esteem.

### Methodological Quality

The quality of the included studies varied. According to the calculated quality assessment score (QAS), none of the studies were rated as being of poor quality, 6 studies were rated fair, 7 were rated good, and 1 was rated excellent. The mean QAS for the included studies was 19.6. Studies scored particularly poor on the following items: patient blinding (11 of 14), blinding of the outcome assessor (12 of 14), failure to adjust for confounding factors in the analysis (11 of 14), bias due to losses of patients to follow-up (9 of 14), and insufficient power to detect outcomes that are clinically important (6 of 14). Furthermore, in 9 of the 14 studies the randomization method and concealment were not described adequately. Because participants in Web-based research are not representative of the whole patient population (in this case through a selection process of only Internet users), the external validity of all studies was rated poor.

### Effectiveness of Web-based Interventions (Web-based Interventions versus Usual Care)

#### Empowerment

Empowerment was measured in 2 studies with the Diabetes Empowerment Scale (DES). Homko et al [55] examined the effectiveness of an Internet-based telemedicine system that was aimed at self-management of underserved women with diabetes mellitus. Empowerment was assessed at baseline and at 37 weeks of gestation. The control group received paper logbooks, which served as a sham intervention, and in which women could monitor their blood glucose levels. In the study of Hill et al [53], the influence of an online intervention containing self-help support groups and Web-based educational tools on empowerment was examined among chronically ill women.

Because the study of Hill et al included only the 10-item “Setting and Achieving Goals” subscale from the DES, our comparison of the two studies was based on the results of this subscale alone.

Based on the GRADE approach, we downgraded the level of evidence two levels, that is, from high to low, on basis of the studies’ limitations and imprecision of the results (Table 2).

**Figure 2:**
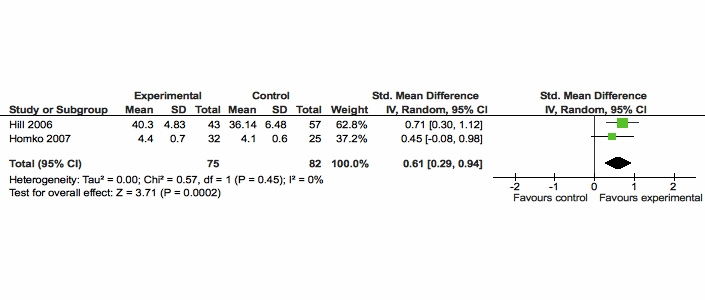
Comparison of Web-based interventions versus usual care for the outcome empowerment

Therefore, based on 2 RCTs (combined n = 157) our results, shown in Figure 2, indicate low quality evidence that Web-based interventions had a significant positive effect on empowerment measured with the DES scale. Figure 2 shows that the SMD for these studies was 0.61 (95% CI 0.29 - 0.94). In Figure 2, the green squares indicate the individual study’s effect sizes, and the black diamond represents the pooled effect of the combined studies.

#### Self-efficacy, Disease-specific

Of the 14 included studies, 9 studies provided sufficient data for calculation of an SMD for disease-specific self-efficacy outcomes. Cousineau et al [51] examined the effects of an online psycho-educational support program for infertile women. In this trial, where 190 females were recruited from US fertility centers, a trend was observed for improvement of self-efficacy levels among the women exposed to the online program compared with the controls (*P* = .07). Hirai et al [54] found that use of an Internet-based self-change program for traumatic event-related fear, distress, and maladaptive coping increased self-efficacy significantly (*P* < .01). In three large and relatively high quality studies (the average Downs and Black score was 21) by Lorig et al [56-58], disease-specific self-efficacy was significantly increased after use of online interventions compared with usual care. These studies contained interventions that dealt with management of heart-lung disease and type 2 diabetes (*P* = .06) [57], arthritis or fibromyalgia (*P* = .01) [58], and back pain (*P* = .02) [56]. Although the duration of the interventions used in these studies was approximately 6 weeks, improvements in self-efficacy remained even after 1 year of follow-up. In a study by Man et al [45], in which people with acquired brain injury (ABI) were able to follow a tele-analogy-based problem-solving program, it was found that after 8 weeks, self-efficacy levels increased more among patients using the program compared with the controls, but this effect was not significant. Ross et al [60] found a trend in improvement of self-efficacy in patients with congestive heart failure who used a Web-based online medical record and educational guide, compared with patients who received usual care (*P* = .08). In a study of Tuil et al [61], patients who had to undergo in vitro fertilization (IVF) were empowered by an interactive website that contained health information, a medical record, and communication possibilities with fellow patients and a physician. No pre/post test differences in IVF-specific self-efficacy were found between the intervention and control group in this study. Finally, a pilot study conducted by Zutz et al [62] found a higher level of exercise-specific self-efficacy after use of an interactive website that was aimed at cardiac rehabilitation. Compared with changes in the control group, this intervention effect was not significant.

According to GRADE guidelines, we downgraded the level of evidence for this outcome by one level from high to moderate based on studies’ limitations (see Table 2).

Visual inspection of the funnel plot on this outcome indicated a possibility of publication bias. Figure 4 shows that small studies, as indicated by the high standard errors [SE] (y-axis), with relatively high effect sizes, as indicated by high SMDs (x-axis), were more present in this analysis than small studies with small positive or negative effects. A possible reason for this is that small and effective (pilot) studies are more likely to be published [45,54,62]. We did not, however, downgrade the quality of evidence on basis of the funnel plot because removing these small studies from the analysis did not result in a change of the pooled estimate.

**Figure 3:**
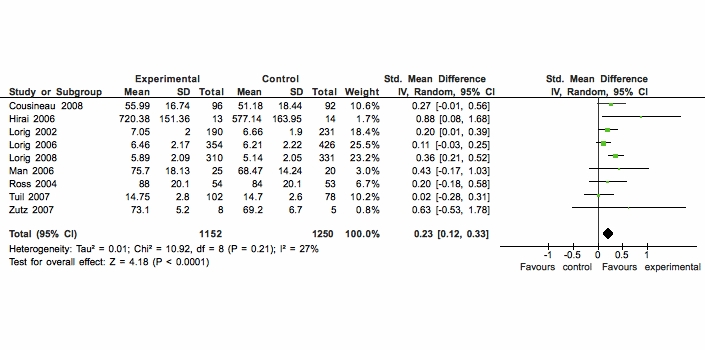
Comparison of Web-based versus usual care for the outcome disease-specific self-efficacy

Therefore, there was moderate quality evidence from 9 studies (combined n = 2402) that Web-based interventions had a significant positive effect on self-efficacy measured with disease-specific self-efficacy scales. Figure 3 shows that the SMD of these 9 studies was 0.23 (95% CI 0.12 - 0.33).

**Figure 4:**
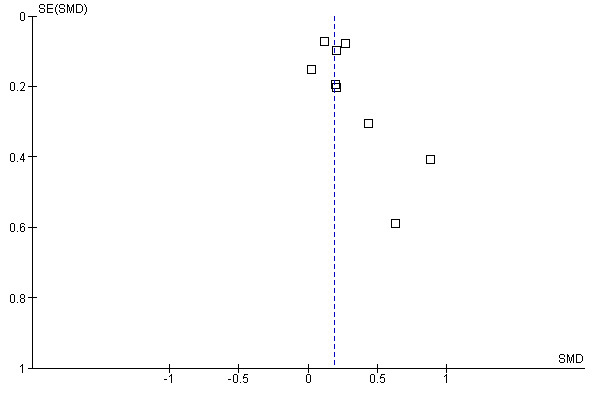
Funnel plot for comparison of Web-based interventions versus usual care for the outcome disease-specific self-efficacy

#### Self-efficacy, General

In three studies, general self-efficacy was measured using general self-efficacy (GSE) scales [64,65]. In the study of Hill et al [53], no significant effect from the computer-based intervention was found on general self-efficacy. Likewise, in the studies of Tuil et al [61] and Zutz et al [62], the Web-based interventions did not have a significant impact on general self-efficacy levels.

We downgraded the level of evidence by two levels, from high to low, based on limitations in the studies and on basis of imprecision of the results (see Table 2).

**Figure 5:**
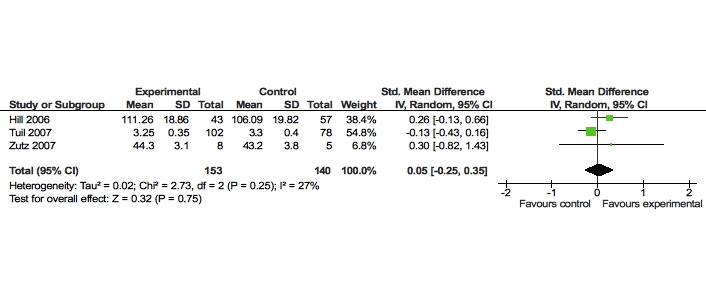
Comparison of Web-based versus usual care for the outcome general self-efficacy

Therefore, based on the GRADE approach, there was low quality evidence from 3 studies (combined n = 293) that there was no statistically significant difference between Web-based interventions and usual care in increasing general self-efficacy. The SMD of these 3 studies was 0.05 (95% CI -0.25 to 0.35). (See Figure 5).

#### Mastery

One study used the Pearlin Mastery Scale [66] to measure the construct mastery, an outcome that is often linked to empowerment. In this RCT by Warmerdam et al [50], two independent Web-based interventions were tested as to their effectiveness to treat adults with depressive symptoms. The first intervention, which lasted 5 weeks, was a problem-solving treatment (PST) based on the idea that by solving people’s practical problems their depressive symptoms will improve. The second intervention, which lasted 12 weeks, was a cognitive behavioral therapy (CBT), aimed at changing people’s cognitive patterns to decrease symptoms of depression. Pre- and post treatment mastery levels between the intervention groups and a control group on a waiting list were compared. Both interventions were found to have had a significant effect on mastery, when compared with the control group. In our analysis, we combined the effects of the two interventions and compared them to the studies’ control group. At 12 weeks follow-up, average mastery levels for both interventions combined was 22.32 (SD 4.17), while the waiting list control group scored 19.37 (SD 3.75). The difference between the control group and the Web-based interventions was significant.

The mean difference [MD] was 2.95 (95% CI 1.66 - 4.24).

Because there was only one study available, according to GRADE there was low quality evidence (one study, n = 263) that Web-based interventions had a significant positive effect on mastery measured with the Pearlin Mastery scale.

#### Self-esteem

Only one study included a measurement of the construct self-esteem, measured with the Rosenberg Self-esteem (RSE) Scale [67]. In the study of Hill et al [53] no significant effects were found for the intervention on self-esteem. The MD was -0.38 (95% CI -2.45 to 1.69).

For this one study (n = 120), based on the GRADE approach, there was low quality evidence that there was no statistically significant difference between Web-based interventions and usual care in increasing self-esteem.

Summarized in Table 2 are the results of the GRADE approach to judging the quality of the evidence of the studies included in this review. Since all included studies were randomized trials, we started with a high quality of evidence and downgraded, if necessary, on basis of the 5 GRADE domains.

To be able to make clinical interpretations of the reported SMDs described above, we reexpressed the pooled SMDs into MDs, by using the technique of back-translation to a familiar instrument. This technique has been described in more detail elsewhere [43]. Table 3 shows the absolute effects (MDs) on the outcomes for which SMDs were calculated, by using back-translation.

**Table 2:** Overall judgment of quality of evidence using the GRADE approach

Outcome Measure	N of Studies	Limitations	Inconsistency	Indirectness	Imprecision	Quality of Evidence
Empowerment	2	Serious^a,b,c^	No serious inconsistency	No serious indirectness	Serious^d^	Low^e^
Self-efficacy (specific)	9	Serious^a,b,c^	No serious inconsistency	No serious indirectness	No serious imprecision	Moderate^f^
Self-efficacy (general)	3	Serious^a,b,c^	No serious inconsistency	No serious indirectness	Serious^d^	Low^e^
Mastery	1	-	-	-	-	Low^g^
Self-esteem	1	-	-	-	-	Low^g^

^a^ Possibility of a lack of allocation concealment

^b^ Lack of blinding

^c^ The majority of the studies did not apply intention-to-treat analyses

^d^ Pooled effect size upper/lower confidence limit crosses 0.5

^e^ Not enough studies available for a funnel plot

^f^ Publication bias is likely, but it does not affect the pooled estimate

^g^ Low quality of evidence on basis of only 1 study available

**Table 3:** Back-translation of SMDs into MDs by using a familiar instrument

Outcome Measure	N of Patients	Mean Follow- up Period	Relative Effect	Chosen Instrument for Back-calculation (Range)	Absolute Effect
Empowerment	157	12 weeks	SMD = 0.61 (95% CI 0.29 - 0.94)	DES [63] (1-5)	MD 0.31 (95% CI 0.15 - 0.47)
Self-efficacy (specific)	2402	23 weeks	SMD = 0.23 (95% CI 0.12 - 0.33)	Self-efficacy Scale, as used by Lorig et al [57] (1-10)	MD 0.42 (95% CI 0.22 - 0.6)
Self-efficacy (general)	293	13 weeks	SMD = 0.05 (95% CI -0.25 to 0.35)	General Self-efficacy Scale Schwarzer [64] (1-5)	MD 0.02 (95% CI -0.1 to 0.14)

### Web-based Interventions Versus Face-to-face Interventions

#### Mastery

Nguyen et al [59] compared an Internet-based dyspnea self-management program for chronic obstructive pulmonary disease (COPD) patients with a face-to-face delivery of the same intervention. In this pilot study, 39 COPD patients were randomized into either one of the two intervention groups. At baseline and after a six-month follow-up period, mastery was measured. Results from this study indicated a slight but not significant advantage for the Internet-based delivery compared with the face-to-face intervention (MD = 1.20, 95% CI -1.73 - 4.13).

Because we found only one study (n = 50) in this category, there is low quality evidence that there is no statistically significant difference between Web-based interventions and face-to-face interventions in increasing mastery.

#### Self-esteem

Gollings et al [52] compared an Internet and face-to-face delivery of a group body image and disordered eating intervention for women. In this 8-week program, participants were able to communicate either online or face-to-face with a therapist that moderated 8 step-by-step group sessions aimed at self-evaluation, managing social pressures and problem solving around weight, and shape and eating issues, for example. Before the intervention and at 8 weeks after the intervention, self-esteem was measured with the RSE in both groups. Although self-esteem improved after both interventions, no significant differences were found between the online and face-to-face delivery (MD = -0.10, 95% CI -0.45 to 0.25).

Again, because we found only one study (n = 39) in this category, there is low quality evidence that there is no statistically significant difference between Web-based interventions and face-to-face interventions in increasing self-esteem (MD = -0.10, 95% CI -0.45 to 0.25).

## Discussion

The Internet revolution and growing need for patient empowerment initiatives has resulted in many Web-based empowerment interventions that have been scientifically evaluated. With this systematic review we intended to gain more insight into the effectiveness of these interventions on empowerment or empowerment-related outcomes.

### Main Findings

In this systematic review, 13 RCTs and 1 quasi-RCT were included. The included studies were clinically heterogeneous regarding included patients, duration and intensity of the intervention, duration of follow-up, and measured outcomes. Statistical pooling was considered to be appropriate in studies measuring the same outcome and comparing the same treatments (either Web-based vs usual care or Web-based vs face-to-face). This resulted in seven comparisons. Statistical pooling within these comparisons showed that Web-based interventions have a significant positive effect on empowerment measured with the DES (2 studies), self-efficacy measured with disease-specific self-efficacy instruments (9 studies), and mastery measured with the Pearlin Mastery Scale (1 study). No significant effects of Web-based interventions were found on self-efficacy measured with general self-efficacy scales (3 studies) and self-esteem measured with the Rosenberg self-esteem scale (1 study). When comparing Web-based interventions with face-to-face deliveries of the same interventions, no statistically significant effect was found in favor of either one of the two deliveries, when the outcome mastery (1 study) or self-esteem (1 study) was measured. Based on the GRADE approach, we found that the evidence for most of the findings described above is of low quality. This means that high quality future research is likely to have an effect on our confidence in the estimate of the effect. The main reason for the low quality of evidence was that many comparisons contained only one study. In the comparisons with more studies available, limitations in study design (lack of blinding, allocation of treatment, not taking into account loss to follow-up) and imprecision of the results, resulted in downgrading the level of evidence.

### Methodological Issues

Although the results of this systematic review indicated that there is some evidence that Web-based interventions are effective in increasing certain empowerment or empowerment-related outcomes, the level of evidence for these effects is rather low, and the results should be interpreted with caution. The basis for the low evidence lies in several methodological issues. First, almost all included studies based their main conclusions on analysis of treatment rather than intention to treat. In this case, results are exposed to a high risk of bias, because characteristics from participants who comply with the treatment may differ from non-participants. This is especially the case in Web-based interventions, where it is known that selection bias is evident, that is, familiarity with the use of computers and the Internet leads to self selection in the use of these technologies [68]. Results of the studies included in this review may thus overrate the effect of the interventions on the patient population as a whole. Moreover, compliance in Web-based research is often found to be low [69], and therefore it seems that Web-based interventions are not suitable for everyone. The issue of low compliance also increases the risk of bias. Other issues that also increase the risk of bias in the studies we found and that should be taken into account involve not adequately describing the randomization method and the lack of patient blinding.

Another concern is the likelihood of publication bias. In the comparison where disease-specific self-efficacy was the outcome, a funnel plot showed some evidence of bias due to publication of smaller and more effective studies or pilot studies [45,54,62]. In the other comparisons and for outcomes represented by only one RCT, many relatively small studies were found, and the choice to publish these studies may have been based on their effectiveness.

Despite these limitations, our meta-analysis included only RCTs or quasi RCTs, which gives our findings a greater robustness than would have been possible if other study designs had been included. Furthermore, by applying the GRADE approach to determine the level of evidence of the effect of an intervention on a set of relevant outcomes, we were able to draw balanced conclusions and give transparency on the basis of how this level of evidence was determined. 

A final point involving the methodology of this review is our choice to statistically pool the results of some of the included studies. Even though pooling included studies that measured the same outcome, these studies were clinically heterogeneous with regard to types of patients, duration and intensity of intervention, and duration of follow-up measurements. It has been argued that in the face of this diversity one should not attempt to perform a meta-analysis [70]. We, however, tried to obviate the clinical heterogeneity by pooling studies that measured the same outcome and by choosing a more conservative random-effects model in our meta-analyses. Also, we think that the general question asked by this review, that is, whether Web-based interventions are effective in increasing patient empowerment, could only be answered by including a broad spectrum of studies where Web-based interventions have been used to date for this purpose. As a result of this choice, caution is advised when interpreting this review in that the results may only apply to specific disease or to specific time frames (eg, short-term vs long-term effects).

### Other Issues

#### Self-efficacy

Statistically significant effectiveness of Web-based interventions on disease-specific self-efficacy was found. On the other hand, no effects were found for general self-efficacy. Because general self-efficacy refers to a broad and stable sense of personal competence, it is possible that Web-based interventions aimed at a specific target patient population are too specific to influence a stable personal characteristic (ie, a trait). Therefore, it has been recommended that for the majority of applications, perceived self-efficacy should be conceptualized in a situation-specific manner [71]. In line with this recommendation, a systematic review of Murray et al [72] found that among children suffering from a chronic disease, self-efficacy was more likely to improve after use of Interactive Health Applications compared with no intervention (SMD = 0.24, 95% CI 0.00 - 0.48).

#### Sustainability of the Effects

From the results of this review, little is known about the sustainability of the effects. In most cases we included data that was measured directly after participants were exposed to the intervention. The effects that are reported, therefore, reflect a direct effect of the intervention. On the other hand, in the studies of Lorig et al [56-58], which measured disease-specific self-efficacy, data was presented only at 6-month or 12-month follow-ups, while the intervention in these studies lasted 6 weeks. Because of the high number of participants included in these studies (total weight in the comparison is 67.1%) there are some signs that the effects of these interventions remain after a longer period of time.

#### Clinical Relevance

We calculated SMDs in this review. This means that the effect sizes presented do not represent certain improvements on specific instruments. To be able to say something about the magnitude of the effect sizes we found, we used back translations of SMDs to a familiar instrument. The results of these back calculations are shown in Table 3. We should, however, realize that these transformations from SMDs to MDs are somewhat arbitrary and should be interpreted with caution. Nevertheless, we found that the statistically significant improvements in empowerment and empowerment-related outcomes were rather small effects: empowerment measured with the DES increased 6.2% after use of the Web-based interventions and self-efficacy measured with disease-specific self-efficacy scales improved 4.2% compared with usual or no care. What the direct or indirect impacts (on clinical outcomes, for example) of these improvements are remains unknown.

### Conclusions

#### Implications for Clinicians

Based on this review, there is some evidence that the Internet can be an effective method to increase patient empowerment. The results from this review show that Web-based interventions can be effective in increasing empowerment among patients who are, for example, suffering from diabetes, depression, infertility, or arthritis. These findings are in line with the growing literature on the effectiveness of eHealth interventions in general, and on outcomes other than patient empowerment [68,72,73]. Clinicians who are interested in empowering their patients are encouraged to refer their patients to Internet empowerment websites, if available and appealing to the patient.

#### Implications for Research

The outcome empowerment usually refers to achieving self-efficacy, mastery, and control. Although many researchers underline that these constructs are closely related to the concept empowerment [33], still much is unclear about how empowerment is defined and how it should be measured. For example, recent work from Aujoulat et al [74] added that empowerment also includes a process of accepting relinquishment of control instead of solely gaining control. Either way, much work lies ahead for researchers in defining and conceptualizing the term empowerment. This will enable combining more unambiguous research outcomes and lead to better insight into the conditions under which, and the individuals for whom, Web-based interventions are effective and how effectiveness can be maximized. Furthermore, future Web-based RCTs aimed at patient empowerment should be conducted on large populations, include the intention-to-treat principle in their analysis [69] and, if applicable, use “sham” website interventions to blind participants from treatment in order to increase the quality of evidence in this field of research. In this perspective, to minimize the risk of bias, researchers are encouraged to consult quality assessment lists (such as the Downs and Black list used in this review) prior to conducting a trial.

## Multimedia Appendix 1

Medline search strategy

## Abbreviations

ABI: acquired brain injury
CBT: cognitive behavioral therapy
COPD: chronic obstructive pulmonary disease
CRQ: Chronic Respiratory Questionnaire
DES: Diabetes Empowerment Scale
GSE: General Self-Efficacy Scale
ICSI: intracytoplasmic sperm injection
IVF: in vitro fertilization
MD: mean difference
PST: problem-solving treatment
QAS: quality assessment scale
RCT: randomized controlled trial
RSE: Rosenberg Self-esteem Scale
SMD: standardized mean difference
WTW: Women-to-Women
WHO: World Health Organization
