# Supplementary material for: Effectiveness of Web-based Interventions on Patient Empowerment: A Systematic Review and Meta-analysis
Source: J Med Internet Res. 2010 Jun 24;12(2):e23. doi: 10.2196/jmir.1286 (PMC2956234; doi:10.2196/jmir.1286)
Supplement: Supplementary file 1 [file jmir_v12i2e23_app1.pdf]

MEDLINE search strategy

1. Self Concept/
2. Self Care/
3. Adaptation, Psychological/
4. Internal-External Control/
5. Power (psychology)/
6. Decision Making/
7. Problem Solving/
8. Health Knowledge, Attitudes, Practice/
9. Patient Participation/
10. Patient Acceptance of Health Care/ (no exp)
11. Patient Satisfaction/
12. Awareness/
13. Health Education/
14. Professional-Patient Relations/
15. OR/1-14
16. Computer Communication Networks/
17. Electronic Mail/
18. Telemedicine/
19. internet
20. web
21. web based
22. www
23. website*
24. online
25. mail*
26. email*
27. chat*
28. blog*
29. OR/19-28
30. AND/ 18,29
31. OR/16,17,30
32. empowerment
33. AND/ 29,32 (NOT Medline)
34. OR/ 31,33
35. AND/ 15,34
36. 35 AND Robinson(2002)

PsycINF search strategy

1. Empowerment/
2. Self Concept/
3. Self Control/
4. Self Efficacy/
5. Self Confidence/
6. Self Esteem/
7. Self Determination/
8. Self Management/
9. Assertiveness/
10. Participation/
11. Involvement/

12. Motivation/
13. Intrinsic Motivation/
14. Power/
15. Decision Making/
16. Problem Solving/
17. Helplessness/
18. Internal External Locus of Control/
19. Coping Behavior/
20. Self Care Skills/
21. OR/ 1-20
22. Computer Applications/
23. Decision Support Systems/
24. Computer Assisted Therapy/
25. Computer Mediated Communication/
26. Internet/
27. Online Social Networks/
28. Online Therapy/
29. Websites/
30. OR/ 22-29
31. AND/ 21,30

EMBASE search strategy

1. Empowerment/
2. Self Concept/
3. Self Care/
4. Behavior Control/
5. Learned Helplessness/
6. Motivation/
7. Powerlessness/
8. Assertiveness/
9. Help Seeking Behavior/
10. Problem Solving/
11. Decision Making/
12. Patient Participation/
13. Awareness/
14. OR/ 1-13 (filter 1985-2007; study design)
15. Internet/
16. Information System/
17. Computer Assisted Therapy/
18. Cybernetics/
19. Computer Network/
20. OR/ 15-19 (filter 1985-2007; study design)
21. AND/ 14,20
